# Functional gastrointestinal diseases and psychological maladjustment, personality traits and quality of life

**DOI:** 10.1186/s12876-018-0760-8

**Published:** 2018-02-27

**Authors:** Nishadi Ranasinghe, Niranga Manjuri Devanarayana, Shaman Rajindrajith, Madusanka S. Perera, Samudu Nishanthinie, Tania Warnakulasuriya, Piyanjali Thamesha de Zoysa

**Affiliations:** 1 Base Hospital Kilinochchi, Kilinochchi, Sri Lanka; 2 Department of Physiology, Faculty of Medicine, University of Kelaniya, Thalagolla Road, Ragama, 11010 Sri Lanka; 3 Department of Paediatrics, Faculty of Medicine, University of Kelaniya, Thalagolla Road, Ragama, 11010 Sri Lanka; 4 Department of Psychological Medicine, Faculty of Medicine, University of Colombo, Kynsey Road, Colombo, 000800 Sri Lanka

**Keywords:** Abdominal pain, Functional gastrointestinal disorder, Healthcare consultation, Health related quality of life, Personality, Psychological maladjustment

## Abstract

**Background:**

Chronic abdominal pain is a common worldwide problem and known to be associated with psychological problems. This study evaluated the association between abdominal pain-predominant functional gastrointestinal disorders (AP-FGIDs), psychological maladjustment and personality traits in adolescents.

**Methods:**

Adolescents aged 13–18 years were recruited from 5 randomly selected schools in Ampara district of Sri Lanka. AP-FGIDs were diagnosed using Rome III criteria. Translated and validated Rome III questionnaire (Child report form), personality questionnaire (PAQ) and PedsQL (Pediatric Quality of Life) inventory were used in data collection. Written consent was obtained from a parent and assent was obtained from every child recruited. The questionnaire was distributed in an examination setting to ensure confidentiality and privacy. Research assistants were present during data collection to assist on any necessary clarifications.

**Results:**

A total of 1697 subjects were recruited [males 779 (45.9%), mean age 15.1 years, SD 1.6 years]. AP-FGIDs were present in 202 (11.9%). Those with AP-FGIDs had significantly higher mean scores for all personality traits (hostility and aggression, negative self-esteem, emotional unresponsiveness, emotional instability and negative world view), except dependency. Affected children had lower scores for all 4 domains of HRQoL (physical, emotional, social and school functioning), compared to controls (*p* < 0.05). When the cut off value for Sri Lankan children (89) was used, 66.3% with AP-FGIDs and 48.2% controls had PAQ scores within that of psychological maladjustment (*p* < 0.001). When the international normative value of 105 was used, these percentages were 27.2% and 14.2% respectively (*p* < 0.0001). The scores obtained for PAQ negatively correlated with scores obtained for HRQoL (r = − 0.52, p < 0.0001). One hundred and seventeen adolescents with AP-FGIDs (57.9%) had sought healthcare for their symptoms. Healthcare consulters had higher PAQ and lower HRQoL scores (*p* < 0.05).

**Conclusions:**

Adolescents with AP-FGIDs have more psychological maladjustment and abnormal personality traits than healthy controls. Affected adolescents with higher psychological maladjustments have lower HRQoL. Greater psychological maladjustment and lower HRQoL are associated with healthcare seeking behaviour in adolescents with AP-FGIDs.

## Background

Abdominal pain-predominant functional gastrointestinal disorders (AP-FGIDs) include irritable bowel syndrome (IBS), functional dyspepsia (FD), functional abdominal pain (FAP) and abdominal migraine (AM) [1]. This group of disorders lead to frequent episodes of abdominal pain or discomfort, which may be associated with distressing bowel related symptoms. Approximately 10–14% of school age children and adolescents around the world suffer from AP-FGIDs and it indicates the extent of the problem [2–4].

Exact pathophysiology of AP-FGIDs is not clear. However, alterations in gut sensitivity and secretomotor functions due to modulation of brain-gut axis by psychological factors such as stress have been suggested as a possible underlying mechanism [5]. The personality of an individual is a significant factor that can influence his/her perception of painful stimuli and the reaction to them. In addition, the presence of psychological maladjustments and certain personality traits such as negative world view are likely to predispose patients to develop psychological co-morbidities such as depression, anxiety and stress. Several studies conducted in adults with AP-FGIDs have reported some abnormal personality traits [6–11]. However, some other adult studies have failed to demonstrate a significant difference in personality traits in affected individuals [12]. Similarly, a previous study conducted in children has also failed to report abnormal mean personality scores in those with recurrent abdominal pain [13]. Therefore, the exact relationship between psychological maladjustment, personality traits and functional gastrointestinal disorders (FGIDs) is not clear. In such a scenario, the main objective of this study was to assess these possible associations.

Health related quality of life (HRQoL) is an important indicator of the impact of a given disease on an individual. Several studies conducted in Sri Lanka and other countries have shown a poor HRQoL indices in adolescents with AP-FGIDs [14–16]. Second objective of this study was to assess the impact of psychological maladjustment and abnormal personality traits on HRQoL of affected adolescents.

According to previous studies, healthcare consultation in children with chronic abdominal pain varies from 28% to 93% [16–22]. Recognised factors that determine healthcare consultation in children with abdominal pain are age of onset, severity, frequency and duration of pain episodes, school absenteeism, sleep interruption, presence of vomiting, abdominal bloating, disruption of normal activities and poor HRQoL [16, 18–20, 23]. The third objective of this study was to assess the impact of psychological maladjustment, personality traits and HRQoL on healthcare consultation of adolescents with AP-FGIDs.

## Methods

### Sample selection

This was a cross sectional survey conducted in the Ampara District of Sri Lanka. Five schools with both female and male students were randomly selected from the list available at the Education Office of the Eastern Province of Sri Lanka. All students from Grade 8 to 13 (aged 13 to 18 years) were recruited.

### Data collection

The questionnaires used in the study were discussed with the relevant Zonal Educational Directors, School Principals, Section Heads and Class Teachers. Following these discussions, permission was obtained to conduct the study.

Information sheets and parental consent forms were handed over to the 1808 eligible study participants two weeks before the data collection. Adolescents took the consent forms home and brought back the consent forms filled and signed by a parent. Reminders were sent to the parents who failed to send back the consent forms. All consent forms were checked and only those who provide consent from a parent to participate were included into the study. None of the parents refused to participate in the study. However, 23 failed to return the parental consent form and were excluded from recruitment. Eighteen study subjects were absent on the date of data collection and were not included. Assent was also obtained from all participants before distribution of the study questionnaires. One participant refused to participate in the study. Sixteen participants had evidence of possible organic disorders causing abdominal pain during the initial clinical evaluation (history and examination). They were also excluded from recruitment and were referred to the local paediatric unit for further evaluation.

Self-administered questionnaires were distributed to 1750 students (who had both written parental consent and given written assent) in an examination setting to ensure privacy and confidentiality. They were collected back after completion on the same day. The research assistants were present at the time of data collection. They explained the purpose of the study to the participants and helped to clarify queries.

All questionnaires were in two main native languages of Sri Lanka (Sinhala and Tamil), and have been pre-tested and validated for Sri Lankan adolescents of the same age group.

### Instruments of data collection

1. Rome III Questionnaire for FGIDs in children (self-report form for children above 10 years) [24] – This questionnaire has been previously translated, validated and used in several studies in Sri Lanka. [2, 3, 16, 25]
2. PedsQL Pediatric Quality of Life Inventory 4.0 (Generic Core Scales) self-report form for teens [26] – This inventory assesses the HRQoL in 4 main domains; physical, emotional, social and school functioning. Higher score indicate better HRQoL. This inventory has been translated and validated by the Mappi Institute. This has been pre-tested and used in several previous Sri Lankan studies. [16, 27, 28]
3. Childhood Personality Assessment questionnaire (PAQ) [29] - This questionnaire measures seven personality traits or dispositions (hostility and aggression; dependence; negative self-esteem; negative self-adequacy; emotional unresponsiveness; emotional instability; and negative worldview). They collectively reflect psychological maladjustment. This questionnaire has been translated and validated for Sri Lankan children of age 12 years. [30]
4. Childhood Healthcare Seeking Behaviour Questionnaire– This has been developed by investigators, pre-tested and used in previous Sri Lankan studies. [16, 20, 31]

For the purpose of this study, AP-FGIDs were diagnosed by using Rome III criteria for FGIDs in children. [1] A clinical evaluation including a detailed history and complete physical examination has been conducted in all adolescents with abdominal pain, to exclude obvious organic pathologies. Adolescents with evidence suggestive of possible organic pathologies during initial evaluation were referred to local paediatric clinics for further evaluation and investigation. Only those with no evidence of organic pathologies and conforming to Rome III criteria were diagnosed as having AP-FGIDs.

### Statistical analysis

Data were analysed using EpiInfo (EpiInfo 6, version 6.04–1996, Centres for Disease Control and Prevention, Atlanta, Georgia, USA and World Health Organization, Geneva, Switzerland). Chi-Squire test was performed using EpiInfo for categorical variables. Multiple logistic regression analysis was performed to assess independent association between factors which were found to have a significant association during univariate analysis. Scores obtained for HRQoL and PAQ were compared using unpaired t-test and Holm-Bonferroni method was used to adjust for multiple comparisons. The effect size was calculated using Cohen D for t-test. Pearson correlation co-efficient was used to correlate scores obtained for HRQoL and PAQ. A *p* value of < 0.05 was considered to be significant.

### Ethical approval

Ethical approval for the study was obtained from the Ethics Review Committee of the Faculty of Medicine, University of Colombo, Sri Lanka.

## Results

### Demographic characteristics of the sample

In this study 1750 questionnaires were distributed. Fifty-three questionnaires did not contain adequate information to diagnose or exclude AP-FGIDs and were excluded from the analysis. Number included in the final analysis was 1697 (97.0%). There were 779 males (45.9%). Age ranged from 13 to 18 years (mean 15.1 years, SD 1.6 years).

### Prevalence of AP-FGIDs

Table 1 shows the prevalence of AP-FGIDs in the study sample. According to the data, IBS is the most prevalent AP-FGID in the sample (4.4%) followed by FAP and AM. FD was seen only in 2.4% of adolescents. Five adolescents fulfil the criteria for both AM and FD and 13 fulfilled the criteria for both AM and IBS. Prevalence of AM was significantly higher in girls (4.5% vs. 1.7% in boys, *p* < 0.001).

**Table 1:** Prevalence of abdominal pain predominant functional gastrointestinal disorders according to sex

	Boys *(n* = 779)		Girls *(n* = 918)		Total *(n* = 1697)	
	*n*	(%)	*n*	(%)	*n*	(%)
Irritable bowel syndrome (IBS) - Total	38	4.9%	36	3.9%	74	4.4%
IBS-diarrhoea predominant	11	1.4%	11	1.2%	22	1.3%
IBS-constipation predominant	5	0.6%	13	1.4%	18	1.1%
IBS-mixed	8	1.0%	5	0.5%	13	0.8%
IBS-untyped	14	1.8%	7	0.8%	21	1.2%
Functional dyspepsia	8	1.0%	17	1.9%	25	1.5%
Abdominal migraine	13	1.7%	41*	4.5%	54	3.2%
Functional abdominal pain	40	5.1%	27	2.9%	67	4.0%
Abdominal pain predominant FGDs-total	80	10.3%	122	13.3%	202	11.9%

FGIDs = functional gastrointestinal disorders

*p = 0.001, chi-square test, girls vs. boys

### Psychological maladjustment and personality traits in adolescents with AP-FGIDs

Table 2 shows the scores obtained for psychological maladjustment and individual personality traits in adolescents with AP-FGIDs. Except for dependence, scores obtained for all other personality traits were significantly higher in adolescents with AP-FGIDs. When Holm-Bonferroni method was used to adjust for multiple comparisons, all personality parameters which were significantly higher in independent sample t test remained to be significant (*p* < 0.05).

**Table 2:** Personality traits in children with abdominal pain-predominant functional gastrointestinal disorders and controls

	IBS (*n* = 74)	FD (*n* = 25)	AM (*n* = 54)	FAP (*n* = 67)	AP-FGIDs total (*n* = 202)	Controls (*n* = 1495)
	Mean (SD)	Mean (SD)	Mean (SD)	Mean (SD)	Mean (SD)	Mean (SD)
Hostility and aggression	13.5 (3.2)*	12.5 (2.7)	13.7 (3.3)*	13.2 (3.9)	13.4 (3.4) **	12.6 (3.4)
Dependence	20.0 (2.5)	19.0 (3.4)	20.0 (3.1)	19.2 (3.1)	19.7 (2.8)	19.7 (2.8)
Negative self-esteem	11.4 (2.9)*	11.8 (2.6)*	11.9 (3.2)**	11.6 (3.6)**	11.6 (3.1) **	10.5 (2.9)
Negative self-adequacy	10.8 (3.4)*	11.0 (3.2)	10.7 (3.6)	11.0 (3.9)**	10.8 (3.5) **	9.9 (3.2)
Emotional unresponsiveness	12.7 (3.2)**	12.8 (3.5)	13.3 (2.8)**	12.4 (3.4)*	12.7 (3.2) **	11.4 (3.1)
Emotional instability	16.9 (3.0)**	16.2 (3.3)	17.4 (3.9)**	16.0 (3.8)	16.7 (3.5) **	15.6 (3.6)
Negative world view	11.5 (3.5)**	11.3 (3.4)	12.1 (3.7)**	10.5 (3.4)	11.2 (3.5) **	10.2 (3.6)
Total personality score	96.9 (13.7)**	94.6 (11.5)	99.0 (14.8)**	94.0 (16.9)*	96.1 (14.7) **	89.8 (14.5)

*p < 0.05, ** *p* < 0.01, compared to controls, unpaired t-test

Since there was a small number of patients with FD, Cohen D for t-test was used to calculate the effect size. Association between FD and hostility and aggression and emotional instability had Cohen D values less than 0.2. All the other comparisons in patients with FD had Cohen D values between 0.2 and 0.5.

**Fig. 1:**
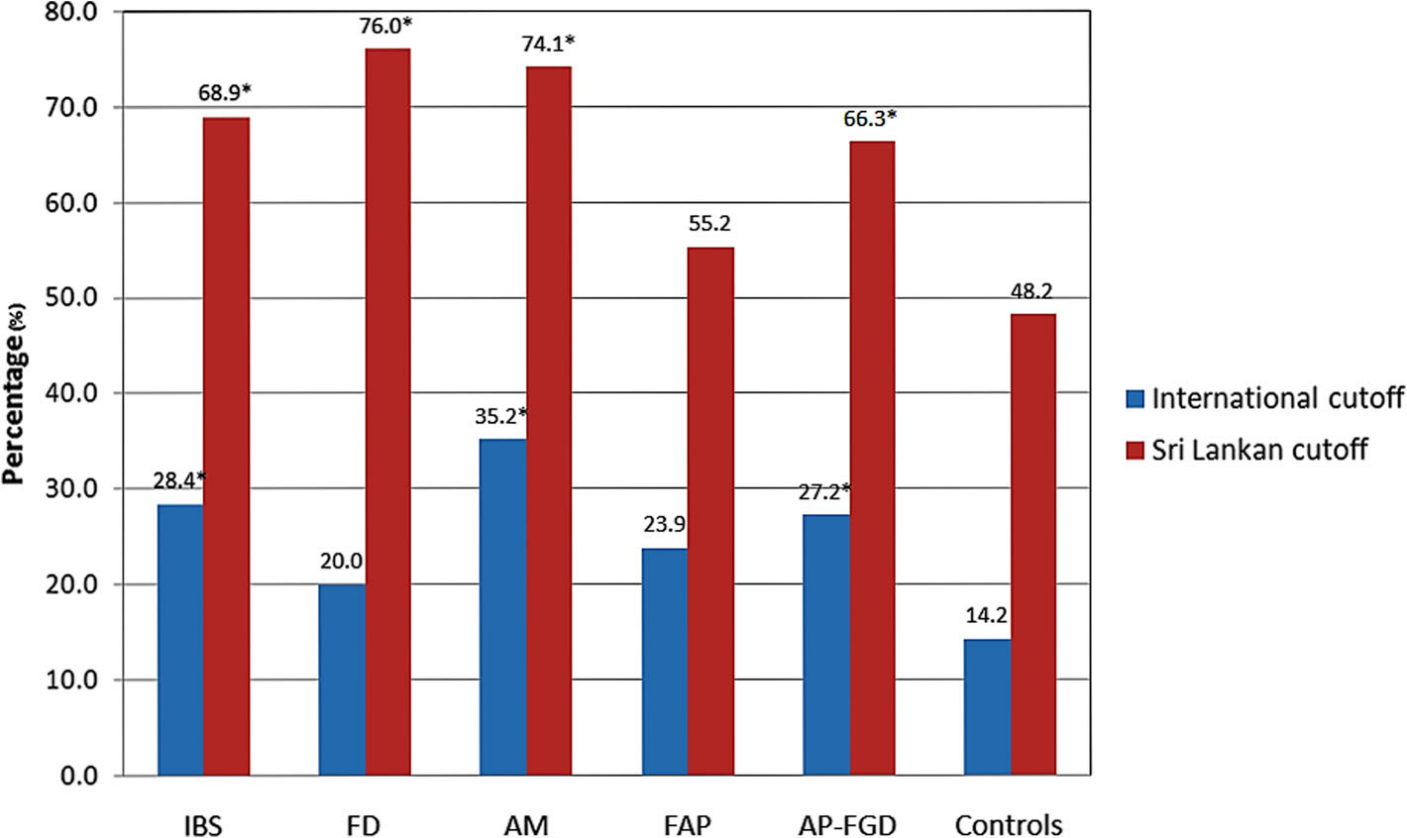
Percentage of subjects with psychological maladjustment according to Sri Lankan and international cut-off values. ^*^p < 0.05, compared to controls (chi-square test)

Figure 1 depicts the percentage of adolescents with all 4 types of AP-FGIDs (and total AP-FGIDs) with psychological maladjustment according to international and Sri Lankan cut-off values. The higher percentage of adolescents with AP-FGIDs had psychological maladjustment irrespective of the cut-off values used.

### HRQoL of adolescents with AP-FGIDs

Table 3 shows scores obtained for HRQoL according to AP-FGIDs. Adolescents with AP-FGIDs have lower scores for all 4 domains of HRQoL compared to the controls. Adolescents with IBS also have lower HRQoL in all 4 domains. However, adolescents with FAP only had the lower score for the physical functioning domain. When Holm-Bonferroni method was used, scores obtained for all domains of HRQoL remained significantly lower in adolescents with AP-FGIDs.

**Table 3:** Health related quality of life scores for children with abdominal pain predominant functional gastrointestinal disorders and controls

	IBS (*n* = 74)	FD (*n* = 25)	AM (*n* = 54)	FAP (*n* = 67)	AP-FGIDs total (*n* = 202)	Controls (*n* = 1495)
	Mean (SD)	Mean (SD)	Mean (SD)	Mean (SD)	Mean (SD)	Mean (SD)
Physical functioning	75.3 (15.1)**	86.1 (11.1)	72.3 (15.9)**	82.2 (12.6)*	78.4 (14.7)**	86.2 (12.7)
Emotional functioning	69.7 (16.6)**	77.4 (14.9)	65.7 (19.1)**	74.4 (17.6)	71.3 (17.5)**	77.4 (17.2)
Social functioning	69.9 (13.7)*	72.6 (13.7)	69.8 (16.3)	71.7 (14.7)	70.6 (14.4)**	73.4 (14.1)
School functioning	70.5 (15.8)**	78.4 (17.2)	66.9 (19.9)**	75.7 (16.9)	72.4 (17.3)**	79.5 (14.9)
Overall quality of life score	71.3 (12.2)**	78.6 (9.4)	68.7 (13.5)**	76.0 (12.6)	73.2 (12.5)**	79.2 (11.6)

*p < 0.05, ** *p* < 0.01, compared to controls, unpaired t-test

**Fig. 2:**
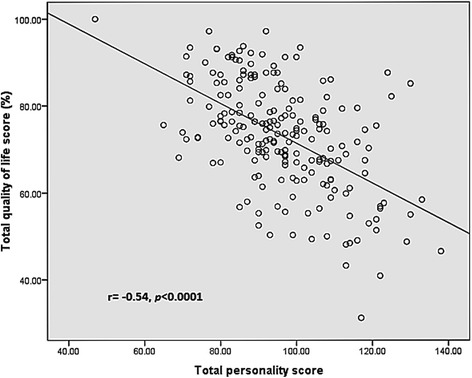
Correlation between scores obtained for health related quality of life and personality in children with abdominal pain-predominant functional gastrointestinal disorders

Figure 2 illustrates the relationship between overall scores obtained for psychological maladjustment and the HRQoL. The scores obtained for psychological maladjustment correlated negatively with the scores obtained for HRQoL.

Total HRQoL score had a weak but significant negative correlations with scores obtained for severity of abdominal pain (r = − 0.23, *p* < 0.001), average duration of abdominal pain episodes in min (r = − 0.22, *p* = 0.002) and duration of AP-FGIDs in months (r = − 0.19, *p* = 0.007) *p* = 0.032). Patients with nausea (70.98 vs. 74.67, *p* = 0.041), vomiting (66.87 vs. 74.01, *p* = 0.01), constipation (67.11 vs. 73.91, *p* = 0.018) and headache (71.18 vs. 75.38, *p* = 0.017) had lower scores for overall quality of life, compared to patients without these symptoms (unpaired t-test).

### Determinants of healthcare seeking

One hundred and seventeen adolescents (57.9%) had sought healthcare for their symptoms. Of them, the majority [89 (76.1%)] have consulted a medical practitioner on an out-patient care basis, while only 28 (23.9%) had received inpatient care.

Healthcare consulters had significantly lower scores for overall HRQoL (71.2 vs. 76.0 in non-consulters) and physical (76.4 vs. 81.2), emotional (68.9 vs. 74.9) and school (69.5 vs. 76.4) functioning domains (*p* < 0.05, independent sample t-test). When Holm-Bonferroni method was used, scores obtained for physical, emotional, school functioning and total HRQoL score remained significantly lower in healthcare consulters.

Scores obtained by different personality traits, such as hostility and aggression (13.9 vs. 12.2 in non-consulters), dependence (20.0 vs. 19.2), negative self-esteem (12.2 vs. 10.8), negative self-adequacy (11.4 vs. 10.2), and emotional instability (17.2 vs. 15.9) were also higher in healthcare consulters than in non-consulters (*p* < 0.05, independent sample t-test). When Holm-Bonferroni method was used, scores obtained for negative self-esteem and total personality score remained significantly higher in healthcare consulters.

When the association between symptom characteristics and healthcare consultation was assessed with multiple logistic regression analysis, presence of bloating (adjusted odds ratio [OR] 2.75, 95% confidence interval [CI] 1.24–6.11, *p* = 0.013), vomiting (adjusted OR 3.54, 95% CI 1.09–11.53, *p* = 0.036) and headache (adjusted OR 1.91, 95% CI 1.03–3.56, *p* = 0.041) were independently associated with healthcare consultation. There was no significant association between healthcare consultation and the socio-demographic, school or family related factors (*p* > 0.05, chi-square test).

## Discussion

AP-FGIDs are a common health problem among 13 to 18 year old children and adolescents in Sri Lanka. In addition, affected teenagers had lower HRQoL, higher psychological maladjustment and more abnormal personality traits. Adolescents with higher PAQ scores, indicating psychological maladjustment, had lower HRQoL. Furthermore, adolescents with higher PAQ and lower HRQoL scores were more likely to seek medical care for their symptoms.

One previous study in Sri Lanka, involving 4 provinces of the country and including the same district, has shown that 12.5% children of 10–16 years had AP-FGIDs [3]. In another study, including 427 children, 13.8% of them were suffering from AP-FGIDs. Both studies have used Rome III criteria to diagnose AP-FGIDs [2]. In the current study, 12.9% adolescents had AP-FGIDs and the data are compatible with previous studies.

According to two previous studies, IBS was the commonest type of AP-FGID in Sri Lanka [2, 3]. Another paediatric study from Colombia has also reported a high prevalence of IBS [32]. The findings of the current study were in accordance with these previous studies. However, a study from Italy using Rome II criteria noted that FD was highly prevalent than other AP-FGIDs in children [33]. It should be noted that, in that study the sample included a significant number of younger children when compared to our sample. Socio-cultural factors are a known set of predisposing factors that determine the prevalence of FGIDs [34]. Differences in socio-cultural factors between the West and the East may have been another factor for differential prevalence of FGIDs between the European samples and our sample.

HRQoL is an important determinant of disease impact on one’s life, especially when there are no reliable biomarkers to quantify disease activity. AP-FGIDs are a group of disorders which gives rise to frequent episodes of abdominal pain and discomfort, and are frequently associated with other symptoms such as abnormal bowel habits, headache, vomiting and nausea. Paucity of effective therapeutic options also increases anxiety and distress in affected children and their parents, contributing further to poor HRQoL. However, few studies have evaluated HRQoL in teenagers with AP-FGIDs so far. A recent school based study, conducted in 10 to 17 years old children, has reported significantly lower quality of school work in children with IBS, aerophagia and cyclic vomiting [35]. Similarly, Varni et al. have evaluated HRQoL using a generic score scale, in children 5 to 18 years in the USA. They reported significantly lower HRQoL in children with IBS in all 4 domains [15]. Another study conducted in high school children in Korea has also reported similar results [36]. A recent Sri Lankan study conducted in the Western Province of Sri Lanka has also noted that teenagers with AP-FGIDs have poor quality of life in all 4 domains [16].

A previous study conducted in Sri Lanka has reported lower quality of life in affected children with bloating, nausea, vomiting and constipation. There was a significant negative correlation between total HRQoL score and severity of abdominal pain and frequency of abdominal pain, severity of dyspepsia and severity of constipation [16]. Similar to this previous study, current study found a significantly lower quality of life in children with nausea, vomiting, constipation and loss of appetite. Furthermore, significant correlations were observed between total HRQoL score and severity of abdominal pain, average duration of pain episodes and duration of AP-FGIDs.

As Sagawa et al.*,* pointed out, poor school functioning is a major problem in children with IBS in Japan, similar to what we had observed in our sample of adolescents with AP-FGIDs [35]. Poor school functioning is likely to results in poor academic performances in affected adolescents, leading perhaps to poor earning capacity as adults. Therefore, it is imperative that health professionals take childhood AP-FGIDs seriously and manage these disorders effectively, in order to prevent long-term adverse outcomes.

PAQ is a self-administered instrument designed to assess seven personality traits [29]. They are hostility and aggression; dependency; negative self-esteem; negative self-adequacy; emotional unresponsiveness; emotional instability; and negative world view. The cut-off values of psychological maladjustment for international use is 105 [37] and in Sri Lanka 89 [30]. The difference in the cut-off scores may suggest cultural differences between Sri Lanka and the USA where child behaviour that may be considered normative in the latter may be considered maladaptive in Sri Lanka.

Using this questionnaire, we found that adolescents with AP-FGIDs have significantly higher scores for psychological maladjustment compared to controls. Adolescents with AP-FGIDs as a group scored higher scores for all abnormal personality traits, except for dependence, than controls. It was of some concern to note that adolescents with IBS, AM, and FAP had more hostility and aggression towards others. In addition, all of them scored higher values for emotional unresponsiveness. Adolescents with these personality traits are likely to have poor social relationships and not many friends. This may partly explain their lower scores in the social domain of HRQoL. In addition, certain personality characteristics such as aggression, hostility and negative world view may predispose adolescents to develop psychological conditions such as depression, anxiety and stress. The only personality trait that was found to be significantly abnormal in adolescents with FD was negative self-esteem. There was a small number of patients with FD in our study sample (*n* = 25). Previous Sri Lankan studies have also reported a low prevalence of FD in adolescents [2, 3]. When effect size was calculated using Cohen D for t-test, a small to medium effect size was observed indicating that this lack of significant difference is likely to be due to small sample size.

Similar to our study, previous adult studies have also reported more negative self-esteem [10] and aggression [7] in adults with functional gastrointestinal disorders. Other abnormalities observed in personality in adults with AP-FGIDs include detachment, neuroticism, conscientiousness, inferior coping strategies, low level of openness and agreeableness [6–10, 38–41]. In contrast to this, some other studies conducted in adults with FGIDs [12] and children with recurrent abdominal pain [13] have failed to demonstrate significant differences in personality in affected individuals. However, all these previous studies have used different tools to assess personality in their study participants and therefore, a direct comparison was difficult.

The psychological factors have been shown to activate the central part of the brain-gut axis [5]. This leads to several physiological changes including increased vagal output and activation of hypothalamo-pituitary-adrenal (HPA) axis. These will ultimately alter gut motility and sensitivity causing symptoms such as abdominal pain and altered bowel habits [42–50]. These hypotheses at least partly explain the physiological basis for associations between psychological maladjustment, personality traits and AP-FGIDs in adolescents.

In addition, recurrent episodes of pain, which is commonly associated with other symptoms such as disturbed bowel habits, dyspepsia, nausea, vomiting, headache and other painful conditions, and unavailability of effective management options may also have contributed to enhance some personality characteristics such as low self-esteem, self-inadequacy and negative world view present in adolescents with AP-FGIDs. It is also possible these adolescents tend to compare themselves with their peers, who do not have such gastrointestinal issues, and develop a maladaptive view towards society at large.

It was interesting to note that none of the types of AP-FGIDs were associated with dependence. We expected them to be dependent on parents and/or healthcare personnel to make them comfortable, at least during the episodes of pain. However, in contrast, dependence is not a personality characteristic that we observed in our group of adolescents with AP-FGIDs. It is difficult to explain this. Teenagers recruited in the current study had symptoms for a very long time and as time passed they may possibly have understood the nature of the disorder, learned that little help can be obtained from family members or healthcare personnel and have become more independent. In contrast, this lack of difference may be due to the reluctance of affected adolescents to admit their dependence, even in an anonymous and confidential questionnaire. Further studies with simultaneous parental questionnaires would be useful to assess the exact nature of the dependence of affected adolescents.

We found a negative correlation between total scores obtained for PAQ and HRQoL. It suggests that psychological maladjustment affects the quality of life of adolescents with AP-FGIDs. This maybe because these adolescents relate -to and view the world in an unrealistic manner which makes them consider the world/others/themselves as unwholesome. Further, affected adolescents with psychological maladjustment may perhaps have more severe symptoms. Psychological adaptations can also influence how symptoms are experienced and interpreted by affected individuals and hence the HRQoL.

In this study, nearly 58% of adolescents have seen a medical practitioner for abdominal pain and related symptoms. Previous studies have reported healthcare consultation rates varying from 28 to 70% of Sri Lankan children [16, 20]. A significant proportion of them were hospitalized due to their symptoms, indicating possible severe symptom exacerbations. However, it is vital to note that the majority of them were treated on an outpatient basis which is consistent with previous studies [16, 20]. Therefore, it is imperative to educate general practitioners and medical officers working in out-patient clinics, on investigation and management of adolescents with AP-FGIDs.

The presence of more psychological maladjustment as well as certain personality traits were associated with higher healthcare consultation. Hence, hostility towards others, emotional instability, negative self-esteem, negative self-adequacy and higher overall psychological maladjustment showed a strong predilection for healthcare seeking behaviour patterns. These personality traits are possibly associated with higher emotional instability and more severe symptoms which lead to more healthcare seeking. The literature regarding AP-FGIDs in adolescents and psychological maladjustment and personality traits is rather sparse and therefore we could not make a worthwhile comparison. However, this novel finding demands the attention of paediatricians and healthcare providers.

It is imperative to note that HRQoL has a significant effect on healthcare consultation. Adolescents with poor HRQoL scores sought more medical care for their symptoms than others. This is especially true when they have lower scores for school, physical and emotional functioning domains. HRQoL is an indirect indicator of the severity of a disease, especially when there are no reliable biological markers, as is the case in AP-FGIDs.

In this study, symptoms independently associated with healthcare consultation in children with AP-FGIDs were bloating, vomiting and headache. Similar to the current study, a previous study conducted in adolescents with AP-FGIDs in Sri Lanka reported an association between vomiting and bloating and healthcare consultation [16]. In this study we did not find a significant independent association between healthcare consultation and socio-demographic, family and school related factors and other clinical characteristics, similar to what previously reported from Sri Lanka [16]. However, previous studies conducted in children with abdominal pain have reported age of onset, severity, frequency and duration of pain episodes, school absenteeism, sleep interruption and disruption of normal activities as important determinants of healthcare consultation [18–20, 23].

This study has several strengths. A large sample size and using questionnaires translated to native languages and validated for Sri Lankan adolescents to diagnose AP-FGIDs and to assess psychological maladjustment, personality traits and quality of life, have increased the robustness of our results. Adolescents with abdominal pain underwent clinical evaluations which included a detailed history and a complete physical examination. Those with evidence of organic pathologies were referred for further investigation in the local paediatric outpatient clinic. This procedure has helped to exclude a majority of adolescents with underlying organic disorders. We collected data using self-administered questionnaires and perhaps the main limitation of our study is recall bias. In addition, when we use the Sri Lankan cut-off value for psychological maladjustment, nearly half of the controls in our sample had psychological maladjustment, but when we apply the international cut-off value it was only 14.2%. This poses a question on the validity of the cut-off value previously available for Sri Lankan children. We feel that the normative data for Sri Lankan children need to be re-calculated using a larger sample of children from different locations of the country.

## Conclusions

The adolescents with AP-FGIDs have more psychological maladjustment and higher PAQ scores indicating abnormal personality traits such as hostility and aggression, negative self-esteem, negative self-adequacy, emotional unresponsiveness, emotional instability and negative world view. In addition, affected adolescents have a poor HRQoL. Adolescents with higher psychological maladjustments had lower HRQoL. Higher psychological maladjustment, some abnormal personality traits and lower HRQoL are associated with higher healthcare seeking behaviour. Therefore, including personality and HRQoL assessment is useful in the clinical evaluation of affected adolescents with frequent healthcare seeking.

## Abbreviations

AM: Abdominal migraine
AP-FGIDs: Abdominal pain predominant functional gastrointestinal disorders
FAP: Functional abdominal pain
FD: Functional dyspepsia
FGIDs: Functional gastrointestinal disorders
HRQoL: Health related quality of life
IBS: Irritable bowel syndrome
